# Engineering *Corynebacterium glutamicum* for de novo production of 2-phenylethanol from lignocellulosic biomass hydrolysate

**DOI:** 10.1186/s13068-023-02327-x

**Published:** 2023-05-04

**Authors:** Nianqing Zhu, Wenjing Xia, Guanglu Wang, Yuhe Song, Xinxing Gao, Jilei Liang, Yan Wang

**Affiliations:** 1 Jiangsu Key Laboratory of Chiral Pharmaceuticals Biosynthesis, College of Pharmacy and Chemistry & Chemical Engineering, Taizhou University, Taizhou, 225300 Jiangsu People’s Republic of China; 2 School of Chemistry and Biological Engineering, Nanjing Normal University Taizhou College, Taizhou, 225300 Jiangsu People’s Republic of China; 3 Laboratory of Biotransformation and Biocatalysis, School of Food and Biological Engineering, Zhengzhou University of Light Industry, Zhengzhou, Henan 450000 People’s Republic of China

**Keywords:** *Corynebacterium glutamicum*, 2-Phenylethanol, Lignocellulosic biomass, Xylose, De novo

## Abstract

**Background:**

2-Phenylethanol is a specific aromatic alcohol with a rose-like smell, which has been widely used in the cosmetic and food industries. At present, 2-phenylethanol is mainly produced by chemical synthesis. The preference of consumers for “natural” products and the demand for environmental-friendly processes have promoted biotechnological processes for 2-phenylethanol production. Yet, high 2-phenylethanol cytotoxicity remains an issue during the bioproduction process.

**Results:**

*Corynebacterium glutamicum* with inherent tolerance to aromatic compounds was modified for the production of 2-phenylethanol from glucose and xylose. The sensitivity of *C. glutamicum* to 2-phenylethanol toxicity revealed that this host was more tolerant than *Escherichia coli*. Introduction of a heterologous Ehrlich pathway into the evolved phenylalanine-producing *C. glutamicum* CALE1 achieved 2-phenylethanol production, while combined expression of the *aro10*. Encoding 2-ketoisovalerate decarboxylase originating from *Saccharomyces cerevisiae* and the *yahK* encoding alcohol dehydrogenase originating from *E. coli* was shown to be the most efficient. Furthermore, overexpression of key genes (*aroG*^fbr^, *pheA*^fbr^, *aroA*, *ppsA* and *tkt*) involved in the phenylpyruvate pathway increased 2-phenylethanol titer to 3.23 g/L with a yield of 0.05 g/g glucose. After introducing a xylose assimilation pathway from *Xanthomonas campestris* and a xylose transporter from *E. coli*, 3.55 g/L 2-phenylethanol was produced by the engineered strain CGPE15 with a yield of 0.06 g/g xylose, which was 10% higher than that with glucose. This engineered strain CGPE15 also accumulated 3.28 g/L 2-phenylethanol from stalk hydrolysate.

**Conclusions:**

In this study, we established and validated an efficient *C. glutamicum* strain for the de novo production of 2-phenylethanol from corn stalk hydrolysate. This work supplied a promising route for commodity 2-phenylethanol bioproduction from nonfood lignocellulosic feedstock.

**Graphical Abstract:**

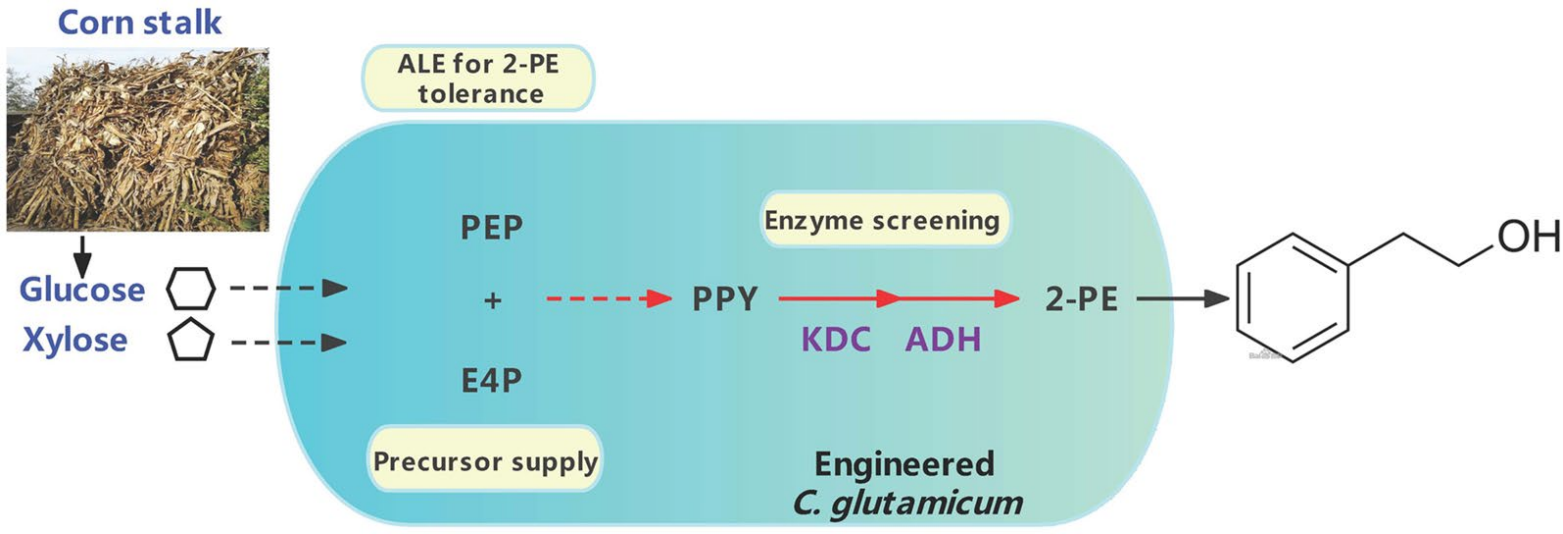

**Supplementary Information:**

The online version contains supplementary material available at 10.1186/s13068-023-02327-x.

## Introduction

Aromatic compounds are a very important class of fine chemicals with a broad market prospect. For example, 2-phenylethanol represents a specific aromatic alcohol with a rose-like smell utilized as a flavor ingredient in the cosmetic and food industries [1]. Natural 2-phenylethanol (about US$ 1000 per kilo) is traditionally extracted from the flower essential oils, such as rose essential oil, lily essential oil, and jasmine essential oil [2]. However, the low concentration of 2-phenylethanol in these plants makes the extraction process costly, thereby restricting the availability. At present, 2-phenylethanol is also produced by chemical synthesis with advantages in yield and cost (about US$ 5 per kilo) [2]; yet, this method requires high temperature since some toxic by-products seriously lower the product quality [3]. The US and European food agencies stipulate that flavors obtained from the biotechnological process are regarded as “natural” products if the used feedstocks are of natural origin [1, 4]. Consumers' preference for “natural” products and the demand for environment-friendly processes have promoted a biotechnological process for 2-phenylethanol production, which is at present considered the most commercially viable route.

2-Phenylethanol can be transformed from L-phenylalanine via the Ehrlich pathway and the styrene-derived pathway using two main types of microbial cell factories (including *Saccharomyces cerevisiae* and *Escherichia coli*) [5–7]. Ehrlich pathway is the most important pathway in which L-phenylalanine is converted to 2-phenylethanol through a three-step enzymatic reaction including transamination, decarboxylation, and reduction. After introducing a self-sufficient cofactor system, the maximum conversion ratios of 95% and 88% were achieved for *S. cerevisiae* and *E. coli*, respectively [7, 8]. Moreover, Machas et al*.* found that the styrene-derived pathway for producing 2-phenylethanol from L-phenylalanine through deamination, decarboxylation, epoxidation, isomerization, and reduction is more efficient and exhibits higher productivity compared to the Ehrlich pathway [9].

Several microorganisms could naturally produce 2-phenylethanol from cheaper carbon substrate such as glucose but at relatively low concentration [10, 11]. Thus, metabolic engineering strategies were made to de novo synthesize 2-phenylethanol from cheaper substrate, which represented a more economic approach. The engineered *S. cerevisiae* with connection of central carbon metabolism and aromatic amino acids metabolism produced 13 mM 2-phenylethanol with a yield of 0.113 mol/mol [5]. To increase 2-phenylethanol productivity, the fast-growing *E. coli* was also engineered as host for de novo synthesis. Kang et al*.* constructed the Ehrlich pathway and overexpressed the phenylpyruvate pathway in *E. coli*, and the engineered strain could produce 0.29 g/L of 2-phenylethanol from glucose [12]. In comparison, another engineered L-phenylalanine producing *E. coli* with the overexpression of styrene-derived artificial cascades produced up to 1.82 g/L of 2-phenylethanol from glucose [9]. Although various strategies were developed, the concentration is still insufficient for industrial production. One of the bottlenecks for bio-production of 2-phenylethanol is cytotoxicity. The hydrophobic nature of 2-phenylethanol led to collapse of the transmembrane gradient and disruption of the cell membrane, which, in turn, decreased cell viability [1, 13]. In order to reduce the cytotoxicity and increase yield, 2-phenylethanol was extracted from the fermentation broth by in situ product removal (ISPR) technologies [1]. With biodiesel as the extractant, high-level 2-phenylethanol production of 9.1 g/L was achieved from glycerol or glucose [6]. Alternatively, biosynthesis of 2-phenylethanol from nonfood carbohydrate feedstocks with robust and tolerant microorganisms without additional extractants could be a more economical strategy.

*Corynebacterium glutamicum* is a rapidly growing facultatively anaerobic, Gram-positive bacterium that has been utilized for large-scale industrial production of a variety of amino acids. The development of metabolic engineering for *C. glutamicum* has increased the spectrum of products produced by this host strain [14]. For the past years, the product range of *C. glutamicum* has been extended to a wide variety of alcohols due to its pronounced resistance [15, 16]. *C. glutamicum* was found to possess better tolerance to alcohols with longer alkyl chains than *E. coli*, such as isobutanol and pentanol isomers [16, 17]. Besides alcohols, *C. glutamicum* was innately tolerant to aromatic compounds and toxic inhibitors derived from lignocellulose biomass hydrolysates, thus these hydrolysates could be directly utilized as feedstock without detoxification [18–20]. Recently, *C. glutamicum* was evolved into a promising host strain for the bioproduction of aromatic chemicals involved in the shikimate pathway, including hydroxybenzoic acids and protocatechuic acid, by inactivating its natural catabolic pathways for aromatic compounds [21, 22]. In particular, *C. glutamicum* exhibited a high tolerance to phenol, catechol and benzoic acid, thus it was engineered for high-level production of muconic acid from lignin [23].

**Fig. 1:**
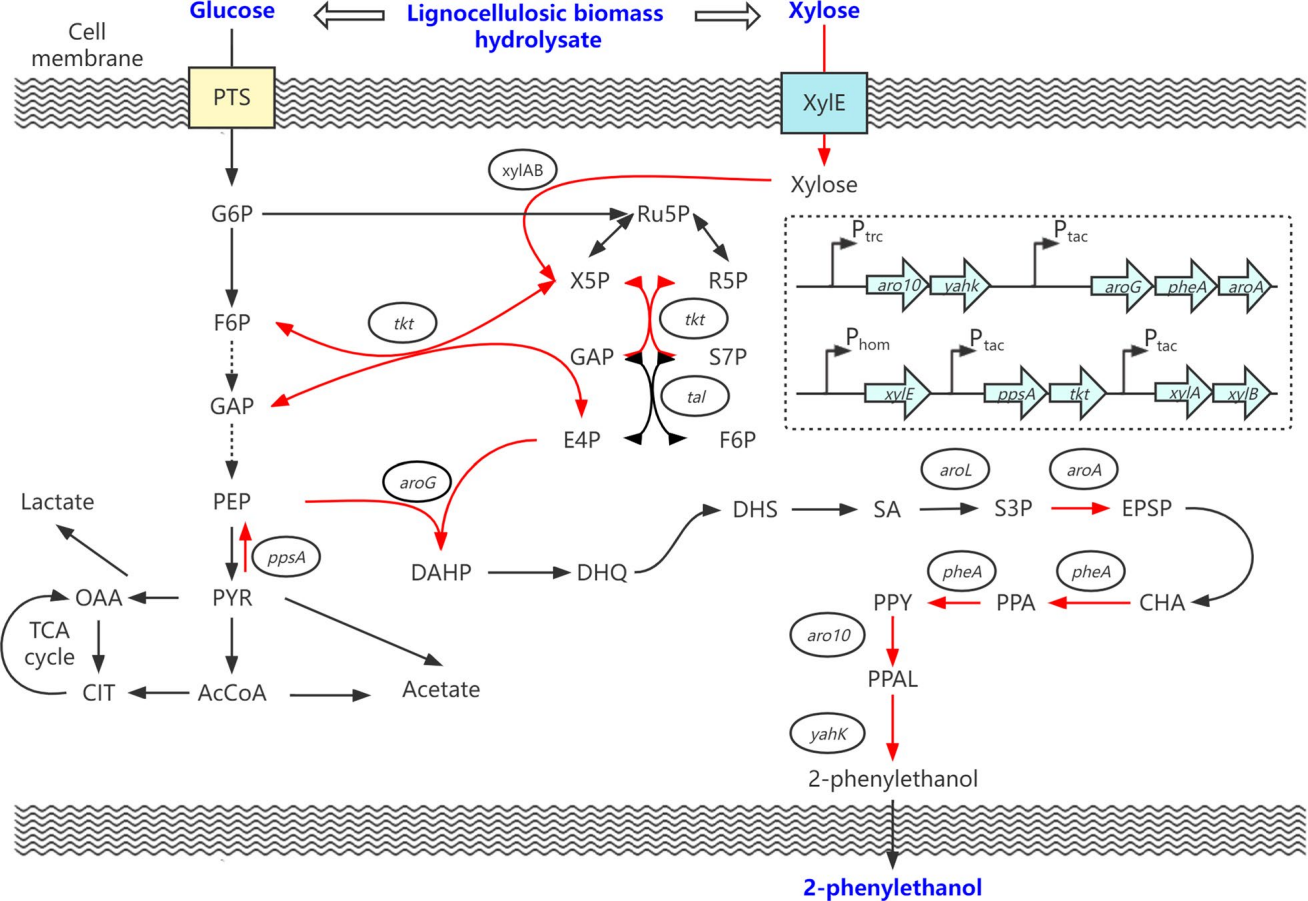
The engineering strategies for de novo production of 2-phenylethanol by *C. glutamicum*. The red arrows indicated that pathways were overexpressed. Metabolites: G6P, glucose-6-phosphate; F6P, fructose-6-phosphate; GAP, glyceraldehyde-3-phosphate;PEP, phosphoenolpyruvate; PYR, pyruvate; OAA, oxaloacetate; CIT, citrate; AcCoA, Acetyl-CoA; Ru5P, ribulose-5P; X5P, xylulose-5P; R5P, ribose-5P; S7P, sedoheptulose‑7‑phosphate; E4P, erythrose‑4‑phosphate; DAHP, 3-deoxy-D-arabinoheptulosonate 7-phosphate; DHQ, 3-dehydroquinate; DHS, 3-dehydroshikimate; SA, shikimate; S3P, Shikimate-3-phosphate; EPSP, 5-enolpyruvyl shikimate 3-phosphate; CHA, chorismate; PPA, prephenate; PPY, phenylpyruvate; PPAL, phenylacetaldehyde. Genes/enzymes: *pts*, phosphoenolpyruvate phosphotransferase system; *xylE*, xylose transporter; *xylA* xylose isomerase; *xylB*, xylulokinase; *tkt*, transketolase; *tal*, transaldolase; *aroG*, DAHP synthase; *ppsA*, phosphoenolpyruvate synthase; *aroL*, shikimate kinase II; *aroA*, 5-enolpyruvylshikimate-3-phosphate synthetase; *pheA*, chorismate mutase-prephenate dehydratase; *aro10*, 2-ketoisovalerate decarboxylase; *yahK*, alcohol dehydrogenase

In this study, the *C. glutamicum* ATCC 21420 was rationally engineered to produce 2-phenylethanol (Fig. 1). *C. glutamicum* was shown to be a suitable host strain for 2-phenylethanol production through analysis of this host’s sensitivity, and two more tolerant strains were obtained by adaptive laboratory evolution. The mutations in the evolved strains were identified by whole-genome resequencing. Several 2-keto acid decarboxylases (KDC) and alcohol dehydrogenases (ADH) were introduced and screened for efficient conversion of phenylpyruvate to 2-phenylethanol. Then, the key genes of the phenylpyruvate pathway were overexpressed to drive more carbon flux to phenylpyruvate. Finally, the feasibility of 2-phenylethanol production from lignocellulosic feedstock with engineered *C. glutamicum* was evaluated.

## Materials and methods

### Bacterial strains, plasmids, and culture media

All bacterial strains, their relevant characteristics, and sources are shown in Table 1. During the process of plasmid construction, *E. coli* JM110 and DH5α used for the gene cloning were cultured with LB medium containing appropriate concentrations of antibiotics (25 mg/L chloramphenicol; 50 mg/L kanamycin). Transferring plasmid DNA into *C. glutamicum* strain was performed by electroporation, and the recombinant clones were screened on brain heart infusion sorbitol (BHIS) agar supplemented with appropriate concentrations of antibiotics (10 mg/L chloramphenicol; 25 mg/L kanamycin). Shanghai Jiuqian Chemical Co., China, provided the detoxified corn stalk hydrolysate, which was prepared by enzymatic hydrolysis. The sterilized corn stalk hydrolysate (115 °C/15 min) contained 148.7 g/L glucose, 67.3 g/L xylose, 0.06 g/L furfural and 0.18 g/L phenolics.

**Table 1:** Bacterial strains and plasmids used in this study

Strains/plasmids	Relevant characteristics	References
Strains		
ATCC13032	*C. glutamicum* wild-type, biotin auxotrophic	ATCC^a^
ATCC21420	*C. glutamicum*, L-phenylalanine producing strain	ATCC^a^
CALE1	2-phenylethanol tolerant strain derived from ATCC 21420	This study
CALE2	2-phenylethanol tolerant strain derived from ATCC 21420	This study
CGPE1	CALE1 (pEC-*aro10yjgB*)	This study
CGPE2	CALE1 (pEC-*pmkdcyjgB*)	This study
CGPE3	CALE1 (pEC-*ipdcyjgB*)	This study
CGPE4	CALE1 (pEC-*aro10yahK*)	This study
CGPE5	CALE1 (pEC-*pmkdcyahK*)	This study
CGPE6	CALE1 (pEC-*ipdcyahK*)	This study
CGPE7	CALE1 (pEC-*aro10adhA*)	This study
CGPE8	CALE1 (pEC-*pmkdcadhA*)	This study
CGPE9	CALE1 (pEC-*ipdcadhA*)	This study
CGPE10	CALE1 (pEC-*aro10yahK*-*aroG*^fbr^*pheA*^fbr^)	This study
CGPE11	CALE1 (pEC-*aro10yahK*-*aroHpheA*^fbr^)	This study
CGPE12	CALE1 (pEC-*aro10yahK*-*aroG*^fbr^*pheA*^fbr^*aroL*)	This study
CGPE13	CALE1 (pEC-*aro10yahK*-*aroG*^fbr^*pheA*^fbr^*aroA*)	This study
CGPE14	CALE1 (pEC-*aro10yahK*-*aroG*^fbr^*pheA*^fbr^*aroA*, pX-*ppsAtkt*)	This study
CGPE15	CALE1 (pEC-*aro10yahK*-*aroG*^fbr^*pheA*^fbr^*aroA*, pX-*P*_*hom*_*xylE-ppsAtkt-xylAB*)	This study
Plasmids		This study
pEC-xk99E	Kan^R^; *C. glutamicum* / *E. coli* shuttle vector (*P*_*trc*_, *lacI*^q^; pGA1, *OriV*_*C.g.*_, *OriV*_*E.c.*_)	[27]
pXMJ19	Cm^R^; *C. glutamicum* / *E. coli* shuttle vector (*P*_*tac*_, *lacI*^q^; pBL1, *OriV*_*C.g.*_, *OriV*_*E.c.*_);	[27]
pEC-*aro10yahK*	For overexpression of *aro10* and* yahK*	This study
pEC-*aro10yjgB*	For overexpression of *aro10* and *yjgB*	This study
pEC-*aro10adhA*	For overexpression of *aro10* and *adhA*	This study
pEC-*pmkdcyahK*	For overexpression of *pmkdc* and* yahK*	This study
pEC-*pmkdcyjgB*	For overexpression of *pmkdc* and *yjgB*	This study
pEC-*pmkdcadhA*	For overexpression of *pmkdc* and* adhA*	This study
pEC-*ipdcyahK*	For overexpression of *ipdc* and* yahK*	This study
pEC-*ipdcyjgB*	For overexpression of *ipdc* and *yjgB*	This study
pEC-*ipdcyahK*	For overexpression of *ipdc* and *adhA*	This study
pEC-*aro10yahK-aroG*^fbr^*pheA*^fbr^	For overexpression of *aro10*, *yahK*, *aroG*^fbr^ and *pheA*^fbr^	This study
pEC-*aro10yahK-aroHpheA*^fbr^	For overexpression of *aro10*, *yahK*, *aroH* and *pheA*^fbr^	This study
pEC-*aro10yahK-aroHpheA*^fbr^*aroL*	For overexpression of *aro10*, *yahK*, *aroG*^fbr^, *pheA*^fbr^ and* aroL*	This study
pEC-*aro10yahK-aroHpheA*^fbr^*aroA*	For overexpression of *aro10*, *yahK*, *aroG*^fbr^, *pheA*^fbr^ and *aroA*	This study
pX-*ppsAtkt*	For overexpression of *ppsA* and* tkt*	This study
pX-*P*_*hom*_*xylE-ppsAtkt-xylAB*	For overexpression of *ppsA*, *tkt*, *xylE* and* xylAB*	This study

^a^ATCC, American type culture collection

### Construction of plasmids and strains

The genes of *aro10*, *pmkdc*, *ipdc*, *yahK*, *yjgB*, *aroH*, *aroG*^fbr^, *pheA*^fbr^, *aroL, ppsA* and *xylE* were synthesized by Genewiz Biotech (Suzhou, China) according to codon preference of *C. glutamicum.* The genes *adhA*, *aroA* and *tkt*, and the *P*_*hom*_ promoter were amplified from the genomic DNA of *C. glutamicum* ATCC 13032 [24]. The *P*_*hom*_ promoter was fused to the *xylE* gene by overlap extension PCR. The *xylAB* genes under *P*_*tac*_ promoter was amplified from the plasmid pX‑xcbAB [25]. DNA oligonucleotides used in this study were listed in Additional file 1: Table S1. The PCR products with suitable Shine Dalgarno sequence and restriction sites were inserted into pEC-XK99E and pXMJ19, yielding a series of pEC-XK99E-based and pXMJ19-based expression plasmids.

### Tolerance test and adaptive laboratory evolution

To investigate 2-phenylethanol tolerance, *C. glutamicum* was cultured with LB medium at 30 °C, while *E. coli* was cultured with LB medium at 37 °C. Briefly, 1 mL of preculture was inoculated into 100 mL LB medium supplemented with different concentrations of 2-phenylethanol (from 1 g/L to 5 g/L). Bacteria were grown with shaking at 200 rpm for 48 h. To further enhance 2-phenylethanol tolerance, *C. glutamicum* ATCC 21420 cells were repeatedly cultivated with LB medium supplemented with 2-phenylethanol using two independent cultures. As the growth of *C. glutamicum* ATCC 21420 was significantly reduced with LB medium containing 3 g/L 2-phenylethanol, this concentration was used for adaptive laboratory evolution. The culture was serially passed every 24 h for 60 times.

### Whole genome sequencing

D*e novo* whole-genome sequencing of C*. glutamicum* ATCC 21420 was performed on the illumine PE150 platform and PacBio Sequel system by Genewiz Biotech (Suzhou, China). The coding genes were annotated with NCBI nr database by Diamond. Then the functions of genes were annotated by GO database, and the pathways were annotated using KEGG database. DNA re-sequencing of CALE1 and CALE2 was performed on the illumina HiseqXten/Novaseq/MGI2000 system by Genewiz Biotech (Suzhou, China). Pipeline of Sentieon (V202112.02) was used to map clean data to reference genome (C*. glutamicum* ATCC 21420), remove duplication and call SNV/InDel. Annotation for SNV/InDel was performed by Annovar (V21 Apr 2018). Breakdancer and CNVnator were used to analyze genomic structure variation.

### Cultivation conditions for 2-phenylethanol production

Single clones of *C. glutamicum* were grown in 10 mL of brain heart infusion (BHI) medium supplemented with appropriate concentrations of antibiotics at 30 °C and 200 rpm. After overnight incubation, cells of the preculture were transferred into 50 mL modified AY medium containing 60 g/L glucose, xylose, or sugar mixture. The modified AY medium contained 7 g/L (NH_4_)_2_SO_4_, 2 g/L urea, 0.5 g/L KH_2_PO_4_, 0.5 g/L MgSO_4_ ∙7 H_2_O, 0.5 g/L K_2_HPO_4_, 6 mg/L FeSO_4_ ∙7 H_2_O, 0.4 mg/L biotin, 6 mg/L MnSO_4_ ∙H_2_O, 0.4 mg/L thiamine, 5 g/L yeast extract, 1 g/L casamino acids, and 21 g/L 3-morpholinopropanesulfonic acid (MOPS) (pH 7.5) [26]. To induce expression, 1.0 mM isopropyl β-D-1-thiogalactopyranoside (IPTG) was added to the cultivation medium. For cultivation with corn stalk hydrolysate as feedstock, the total sugar concentration was adjusted to 60 g/L, which contained 41.3 g/L glucose and 18.7 g/L xylose.

### Analytical methods

The concentrations of L-phenylalanine and 2-phenylethanol were measured by HPLC (HP 1200, Agilent, USA) equipped with a reversed phase column of Poroshell 120 SB-C18 (4.6 × 150 mm, 2.7 micron, Agilent, USA) and a UV absorbance detector (Agilent, USA) at 258 nm with the mobile phase of 70% water and 30% acetonitrile containing 0.1% trifluoroacetic acid at the flow rate of 0.6 mL/min. Organic acids and sugars were measured by HPLC (HP 1200, Agilent, USA) equipped with a cation-exchange column of Aminex HPX-87H (7.8 × 300 mm, BioRad, USA), a refractive index (RI) detector (Agilent, USA), and a UV absorbance detector (Agilent, USA) with the mobile phase of 3 mM sulfuric acid solution at the flow rate of 0.5 mL/min. Acetate, lactate, and shikimate were detected by the UV detector at 210 nm, while the RI detector was used to measure glucose and xylose. Cell growth was monitored by measuring the optical density of cultures at 600 nm (OD_600_), and one unit of optical density corresponded to 0.31 dry cell weight (DCW) (g/L) [27]. The determination of reductive activity of AdhA was performed as described previously [16].

## Results and discussion

### Cytotoxic effect of 2-phenylethanol on *C. glutamicum*

**Fig. 2:**
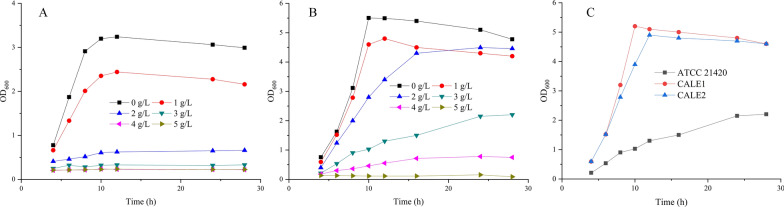
Growth profiles of *E. coli* **A** and *C. glutamicum* ATCC 21420 **B** strains in the presence of different concentrations of 2-phenylethanol, and evolved *C. glutamicum* **C** strains in 3 g/L 2-phenylethanol. *E. coli* was cultured in LB medium at 37 °C, while *C. glutamicum* was cultured in LB medium at 30 °C

The cytotoxicity of higher alcohols is generally far greater than that of alcohols with a shorter alkyl chain such as ethanol [17]. 2-Phenylethanol could severely affect the cellular metabolism of *S. cerevisiae* during its bioproduction, including the reduction of respiratory capacity and restriction of nutrient uptake [28]. In this study, the toxicity of 2-phenylethanol against *C. glutamicum* was examined, and the most widely used chassis *E. coli* was also tested as a control. For the direct comparison, *E. coli* MG1655 and *C. glutamicum* ATCC 21,420 were cultivated in LB medium supplemented with 2-phenylethanol (from 1 g/L to 5 g/L) (Fig. 2A, B). The tolerance of the two hosts to 2-phenylethanol was measured by the relative growth rate after 2-phenylethanol addition. With the addition of 2 g/L 2-phenylethanol, the relative growth rate of *C. glutamicum* was reduced to 61%, while the growth of *E. coli* cells was almost stopped. After adding 3 g/L 2-phenylethanol, the relative growth rate of *C. glutamicum* was 53%; while the 2-phenylethanol was higher than 4 g/L, cell growth was severely inhibited. The results showed that *C. glutamicum* exhibited a better tolerance towards 2-phenylethanol than *E. coli*.

The tolerance mechanisms of *C. glutamicum* to aromatic compounds have been elucidated e.g., complex structures of cell envelope, and effective damage repair and defense mechanisms [29, 30]. These protective mechanisms might be the reason why *C. glutamicum* could stand against alcohols [17, 31]. However, further investigation is required. To further enhance 2-phenylethanol tolerance, adaptive laboratory evolution of *C. glutamicum* ATCC 21420 was performed. The cells were repeatedly cultivated containing 3 g/L 2-phenylethanol using two independent cultures. Two evolved strains, CALE1 and CALE2, were obtained after this selection process, displaying normal growth with 3 g/L 2-phenylethanol (Fig. 2C). The evolved strain CALE1 showed a higher biomass at 20 h and exhibited a higher growth rate than strain CALE2 in the presence of 2-phenylethanol. Analysis of the production performance showed that the phenylpyruvate pathway of the evolved strain was almost unaffected after evolution, as these evolved strains produced comparable concentrations of phenylalanine to *C. glutamicum* ATCC 21420 (Additional file 1: Fig. S1).

### Identification of mutations in the evolved strains by whole-genome resequencing

The de novo whole genome sequence of *C. glutamicum* ATCC 21420 spans 3303123 bp, with a G + C content of 54.3%, containing 3047 predicted coding sequences (GenBank accession No. CP121344). The mutations in CALE1 and CALE2 relative to the parent strain were identified by resequencing their genomes. CALE1 possessed 10 mutations including 9 single nucleotides variants (SNVs) and 1 insertion/deletion (InDel), while CALE2 only possessed 4 SNVs and 2 InDels (Table 2). Of the 10 mutations in CALE1, 8 mutations were found in intragenic regions, while 2 mutations occurred in intergenic regions. The CG_2122 gene, annotated as a mycothiol system anti-sigma-R factor (100% amino acid identity with RshA of *C. glutamicum* ATCC 13032), could not be expressed properly in CALE1 caused by a mutation in the translational start codon of this gene (from ATG to AGG). The *rshA* gene coding for an anti-sigma factor controls the function of the stress-responsive sigma factor SigH in *C. glutamicum* ATCC 13032, and genes related to mycothiol synthesis and recycling, protein degradation, SOS-response to DNA damage and heat stress were upregulated in the *rshA* deletion mutant [32]. Mycothiol was found to contribute significantly to resistance to various poisonous chemicals in *C. glutamicum* [33]. Accordingly, the evolved strain CALE1 might release the repression of SigH and improve the resistance to 2-phenylethanol. A nonsynonymous mutation was found in CG_2149 gene of CALE1, which annotated as a cell wall biosynthesis protein (97.5% amino acid identity with LcpA of *C. glutamicum* ATCC 13032). LcpA is responsible for the transfer of arabinogalactan onto peptidoglycan involved in cell wall biosynthesis [34]*. C. glutamicum* has a complex structured cell wall, which gives it inherent ability to withstand adverse external environments [35]. The amino acid change I136F occurred in the LCP-domain of LcpA, near the position that binding the pyrophosphate head group of the substrate (R138), might affect its catalytic efficiency [34].

**Table 2:** Mutations identified in evolved strains CALE1 and CALE2

Strain	Position	Type	Locus tag	Reference	Mutation	Amino acid change	Description
CALE 1	242720	Nonsynonymous SNV	CG_211	G	T	A104D	Putative membrane protein
	1320434	Intergenic SNV	CG_1192 (upstream dist = 176 bp)	C	T		Copper resistance protein
	1998826	Nonsynonymous SNV	CG_1807	G	A	R370H	Excinuclease ABC subunit A
	2123755	Synonymous SNV	CG_1940	T	C	G194G	ABC transporter permease
	2325348	Nonsynonymous SNV	CG_2122	A	C	M1R	Mycothiol system anti-sigma-R factor
	2351992	Nonsynonymous SNV	CG_2149	A	T	I136F	Cell wall biosynthesis protein
	2404985	Nonsynonymous SNV	CG_2185	G	T	G86V	Sulfur transferase
	2448523	Synonymous SNV	CG_2223	T	C	D96D	Hypothetical protein
	2609654	Synonymous SNV	CG_2388	G	A	R157R	Elongation factor Tu
	649723	Intergenic InDel	CG_566, CG_567 (upstream dist = 76 bp)		G		Putative arsenite efflux pump/IS6 family transposase
CALE 2	242720	Nonsynonymous SNV	CG_211	G	T	A104D	Putative membrane protein
	782863	Synonymous SNV	CG_695	C	T	V18V	IclR family transcriptional regulator PcaR
	1300584	Nonsynonymous SNV	CG_1170	G	A	H339Y	RNA polymerase sigma factor SigA
	1525317	Nonsynonymous SNV	CG_1375	A	G	R75G	Sec-independent protein secretion pathway component
	84404	Intergenic InDel	CG_74 (upstream dist = 30 bp)		T		IS1634 family transposase
	649723	Intergenic InDel	CG_566, CG_567 (upstream dist = 76 bp)		GG		Putative arsenite efflux pump/IS6 family transposase

The evolved strain CALE2 had a nonsynonymous mutation in CG_1170 gene (amino acid change H339Y), which annotated as a primary RNA polymerase sigma factor SigA. This mutation located in the promoter-recognizing 3.0 domain of SigA [36]. Mutations in sigma factors were identified in adaptive evolution and their importance in natural evolution was illustrated [37]. Specially, ethanol tolerance of *E. coli* could be greatly improved by overexpression of randomly mutated main sigma factor RpoD (σ^70^) [38]. In general, mutations in sigma factors could cause global transcription-level alterations [37, 39]. Considering that both evolved strains CALE1 and CALE2 possessed mutations associated with the regulation of sigma factors, the phenotype of improved 2-phenylethanol tolerance might be complex and controlled by a multitude of genes.

### Construction of 2-phenylethanol synthesis pathway

Metabolic engineered *E. coli* could produce aromatic alcohols from glucose by expanding the shikimate pathway with the connection of the Ehrlich pathway [40]. The Ehrlich pathway was also constructed in *C. glutamicum* ATCC 13032 for isobutanol production by introducing a heterologous 2-keto acid decarboxylase; then, 2-phenylethanol was detected as a by-product due to the promiscuous activity of this enzyme [16]. Considering the complex feedback regulation in branched metabolic pathways, the phenylalanine-producing CALE1 strain derived from *C. glutamicum* ATCC21420 was selected as starting strain as it possessed an improved flux in the phenylpyruvate pathway and relatively strong 2-phenylethanol tolerance. Three 2-keto acid decarboxylases (IpdC from *Azospirillum brasilense*, PmKDC from *Proteus mirabilis,* and Aro10 from *S. cerevisiae*) and two alcohol dehydrogenases (YjgB and YahK from *E. coli*) were evaluated in *C. glutamicum* for 2-phenylethanol synthesis, as these enzymes were already successfully used for the production of alcohols [7, 40, 41]. The native NAD-dependent alcohol dehydrogenase AdhA, which appeared to be functional for converting phenylacetaldehyde, was also tested [16].

**Fig. 3:**
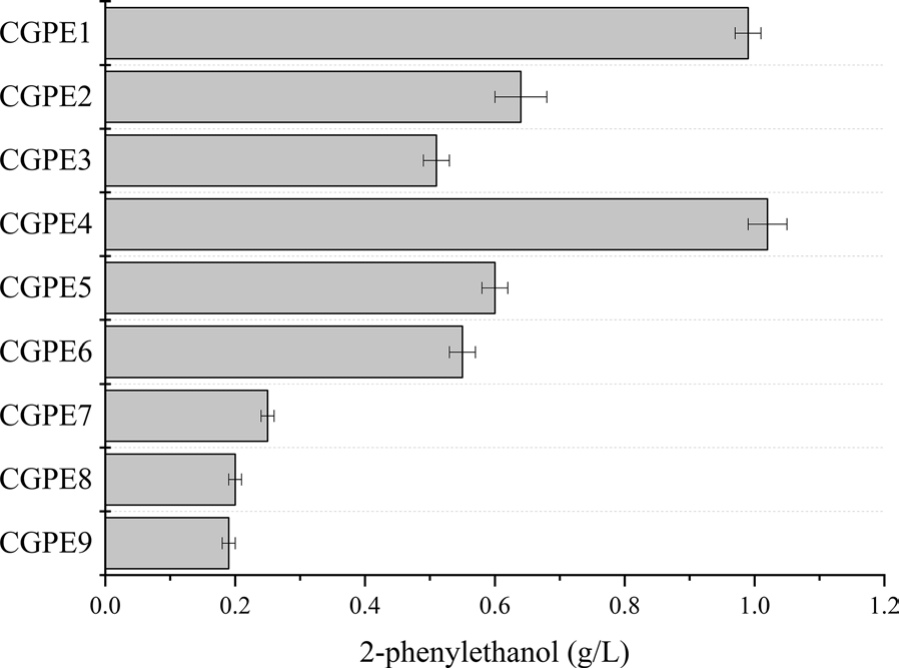
Comparison of 2-phenylethanol production by *C. glutamicum* CALE1 strain with various 2-keto acid decarboxylases and alcohol dehydrogenases. 2-Phenylethanol production was determined after 60 h fermentation in modified AY medium supplemented with 60 g/L glucose. The values were given as the averages and standard deviations of three independent cultures

As shown in Fig. 3, all nine combinations of decarboxylase and dehydrogenase could achieve 2-phenylethanol production, while the choice had a huge impact on the 2-phenylethanol production (from 0.19 to 1.02 g/L). As for decarboxylase, the maximum concentration of 2-phenylethanol with an expression of Aro10 was about 1.8-fold higher than that of PmKDC and IpdC, while there was no difference between PmKDC and IpdC, which could be because Aro10 has a relatively higher affinity to phenylpyruvate (*Km* of 0.10 mM) compared to IpdC (*Km* of 1.08 mM) and PmKDC *(Km* of 0.62 mM) [42, 43]. The Aro10 with excellent property has been used for efficient production of 2-phenylethanol in engineered yeast and *E. coli* [7, 44], and it also performed well in *C. glutamicum*. On the other hand, 2-phenylethanol production via introduction of AdhA was substantially lower in comparison to YahK and YjgB, and there was no significant difference between YahK and YjgB. This might be that AdhA preferred alcohols as substrates, such as ethanol, when *C. glutamicum* was growing on the substrate, despite adequately contributing to producing isobutanol and pentanol [16, 17]. The crude extract enzyme assays were performed to confirm whether AdhA was successfully expressed. For strain CGPE4, the specific activity toward phenylacetaldehyde was 0.02 U/mg, and there was no significant difference compared to the CALE1 strain with only introduction of Aro10 (Additional file 1: Table S2). However, the specific activity toward isobutyraldehyde was detected to be 0.46 U/mg, whereas an activity of 0.05 U/mg was detected without introduction of AdhA (Additional file 1: Table S2). These results demonstrated the efficient expression of AdhA, however, it could not efficiently convert phenylacetaldehyde to 2-phenylethanol. Moreover, the CALE1 strain with only introduction of Aro10 also accumulated 0.26 g/L 2-phenylethanol (Additional file 1: Fig. S2), indicating that endogenous alcohol dehydrogenases of *C. glutamicum* were capable of reducing phenylacetaldehyde to 2-phenylethanol (Additional file 1: Table S2). In the engineered strain CGPE4 with the expression of Aro10 and YahK, maximal 2-phenylethanol production reached 1.02 g/L after 60 h cultivation.

### Driving carbon flux into the phenylpyruvate biosynthesis pathway

The enzymes involved in the synthesis of phenylalanine in *C. glutamicum* are tightly regulated by feedback inhibition. Overexpression of *aroG*^S180F^ encoding a mutated feedback-resistant 3-deoxy-D-arabino-heptulosonate 7-phosphate (DAHP) synthase originating from *E. coli* cumulatively improved carbon flux towards the shikimate pathway [21, 22]. Additionally, co-expression of *aroH* encoding a wild-type DAHP synthase and *pheA*^fbr^ encoding a mutant prephenate dehydratase/chorismite mutase originating from *E. coli* significantly increased phenylalanine production and decreased shikimate production [45]. As 2-phenylethanol shares the precursor with phenylalanine, combinations of *aroG*^fbr^/*aroH* and *pheA*^fbr^ were introduced to evaluate their effects on 2-phenylethanol production. As expected, co-expression of the committed enzymes led to a substantial increase in 2-phenylethanol production, while the combination of *aroG*^fbr^ and *pheA*^fbr^ (CGPE10) exhibited better performance with a production titer of 2.05 g/L compared with the combination of *aroH* and *pheA*^fbr^ (CGPE11) with a production titer of 1.88 g/L (Table 2). Even so, the intermediate shikimate was incidentally accumulated, indicating bottlenecks in the downstream shikimate pathway. A previous study indicated that high-level expression of the downstream genes of shikimate and moderate expression of the upstream genes of shikimate could balance the carbon flux to phenylalanine [27]. The two steps catalyzed by shikimate kinase (*aroL*) and 5-enolpyruvylshikimate-3-phosphate (EPSP) synthase (*aroA*) in *E. coli* [46, 47] and *C. glutamicum* [27] were limiting steps in the synthesis of aromatic amino acids, whose enhanced expression could increase phenylalanine and tyrosine production and reduce intermediates accumulation. Consequently, these genes were individually overexpressed in the CGPE10 strain. As shown in Table 3, overexpression of the native *aroA* (CGPE13) increased 2-phenylethanol production to 2.48 g/L with simultaneously reduced intermediate shikimate accumulation (1.49 g/L). However, enhanced expression of *aroL* from *E. coli* did not obviously affect 2-phenylethanol production (CGPE12), indicating that expression of shikimate kinase gene might not be a rate-limiting factor in our strain background. It might also be the result of the inhibition of shikimate kinase activity by shikimate [46, 48]. The accumulation of shikimate indicated that the downstream pathway of shikimate should be further optimized. In previous reports on 4-hydroxybenzoic acid production with *C. glutamicum* ATCC 13032 and R, simultaneous overexpression of genes *aroCKB*, *aroD*, *aroE* and *aroA* in the shikimate pathway could effectively increase product titers and reduce the formation of shikimate and 3-dehydroshikimate [49, 50]. In particular, introduction of a gene *aroK* encoding shikimate-resistant shikimate kinase from *Methanocaldococcus jannaschii* led to a great decrease in the formation of shikimate and 3-dehydroshikimatae. We thus expected to overcome the accumulation of intermediates during 2-phenylethanol production through enhanced expression of all genes in the shikimate pathway based on the chromosomal integration in future work.

**Table 3:** The production of 2-phenylethanol and other byproducts from glucose by *C. glutamicum* strains

Strain	2-Phenylethanol (g/L)	Shikimate (g/L)	Lactate (g/L)	Acetate(g/L)
CGPE9	1.02 ± 0.01	1.53 ± 0.03	1.99 ± 0.04	3.34 ± 0.05
CGPE10	2.05 ± 0.04	3.15 ± 0.02	1.81 ± 0.02	3.29 ± 0.04
CGPE11	1.88 ± 0.07	3.01 ± 0.04	1.92 ± 0.01	3.31 ± 0.03
CGPE12	2.09 ± 0.09	3.09 ± 0.06	1.85 ± 0.06	3.28 ± 0.07
CGPE13	2.48 ± 0.08	1.49 ± 0.03	1.86 ± 0.02	3.13 ± 0.02
CGPE14	3.23 ± 0.02	1.56 ± 0.02	1.53 ± 0.03	2.61 ± 0.06

The values were given as the averages and standard deviations of three independent cultures. 2-phenylethanol production was determined after 60 h fermentation in modified AY medium supplemented with 60 g/L glucose

Adequate supply of phosphoenolpyruvate (PEP) and erythrose‑4‑phosphate (E4P) is a crucial factor for achieving the maximum carbon flux toward shikimate pathway [27]. Utilization of a non-PTS alternative glucose uptake route and enhanced expression of pentose phosphate pathway genes are effective strategies for the overproduction of shikimate and other aromatics [21, 27, 51, 52]. To increase PEP availability for 2-phenylethanol synthesis, we attempted to block the PTS glucose uptake route by deleting *ptsH* gene; however, the engineering failed due to the infeasibility of chromosome manipulation in this strain background. Alternatively, a PEP synthase from *E. coli* that could covert pyruvate to PEP was introduced into CGPE13 [53–55]. Secondly, the native *tkt* gene was overexpressed to increase the E4P availability. The resulting strain CGPE14 produced 3.23 g/L 2-phenylethanol, increasing by 30% compared to the CGPE13 strain** (**Table 3**).**

### 2-Phenylethanol production from glucose and xylose mixtures

**Fig. 4:**
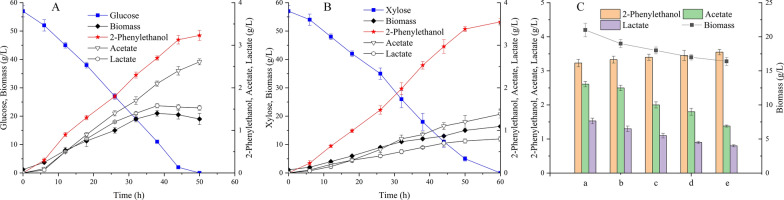
De novo production of 2-phenylethanol by *C. glutamicum* CGPE15 strain with glucose **A**, xylose **B** and different combinations of sugar **C**. a, 60 g/L glucose; b, 40 g/L glucose and 20 g/L xylose; c, 30 g/L glucose and 30 g/L xylose; d, 20 g/L glucose and 40 g/L xylose; e, 60 g/L xylose

Biomass-derived xylose is an economical feedstock for the biotechnological production of value-added chemicals. Usually, *C. glutamicum* is not able to use xylose as the sole carbon source; however, after introducing xylose isomerase (*xylA*) and xylulokinase (*xylB*) from *Xanthomonas campestris*, the engineered strain was able to grow with xylose, showing fast growth rate [56, 57]. This strain simultaneously consumed glucose and xylose mixture with weak carbon catabolite repression. Accordingly, the two genes *xylAB* were heterologously expressed in CGPE14 strain. To facilitate xylose transportation, a pentose transporter gene *xylE* from *E. coli* was further expressed under the weak constitutive *P*_*hom*_ promoter [24]. The xylose utilization ability and the production performance of the resulting strain CGPE15 were evaluated. As shown in Fig. 4, the CGPE15 strain could use xylose as sole carbon and produced 3.55 g/L 2-phenylethanol with a yield of 0.06 g/g xylose, which was 10% higher than that with glucose. Specifically, the accumulation of by-products lactate and acetate derived from pyruvate dramatically decreased when xylose was utilized, indicating a lower pyruvate availability. This might be attributed to the fact that XylE was used for xylose transportation but not the PTS system, which phosphorylated its substrate with phosphoenolpyruvate. This observation was consistent with the previous study in which the by-product lactate accumulation was strongly reduced with xylose fermentation [58]. Notably, the biomass obtained by CGPE15 strain with xylose was 17% lower than that with glucose. This reflected a significant change in the distribution of carbon metabolism in *C. glutamicum*, which was strongly influenced by the substrate*.* The increased TCA flux accompanied by high CO_2_ formation when utilizing xylose was induced by the PPP limitation to supply NADPH, leading to lower biomass yield on xylose [59]. Furthermore, the production of 2-phenylethanol from sugar mixtures of glucose/xylose with different proportions was carried out. The 2-phenylethanol titer and biomass formation obtained for sugar mixture were in between the values obtained for a single substrate. Also, the 2-phenylethanol production increased with the ratio of xylose, along with reduced by-products accumulation. These results suggested that xylose was a better feedstock for 2-phenylethanol production in the CGPE15 strain background.

### 2-Phenylethanol production from plant biomass hydrolysates

**Fig. 5:**
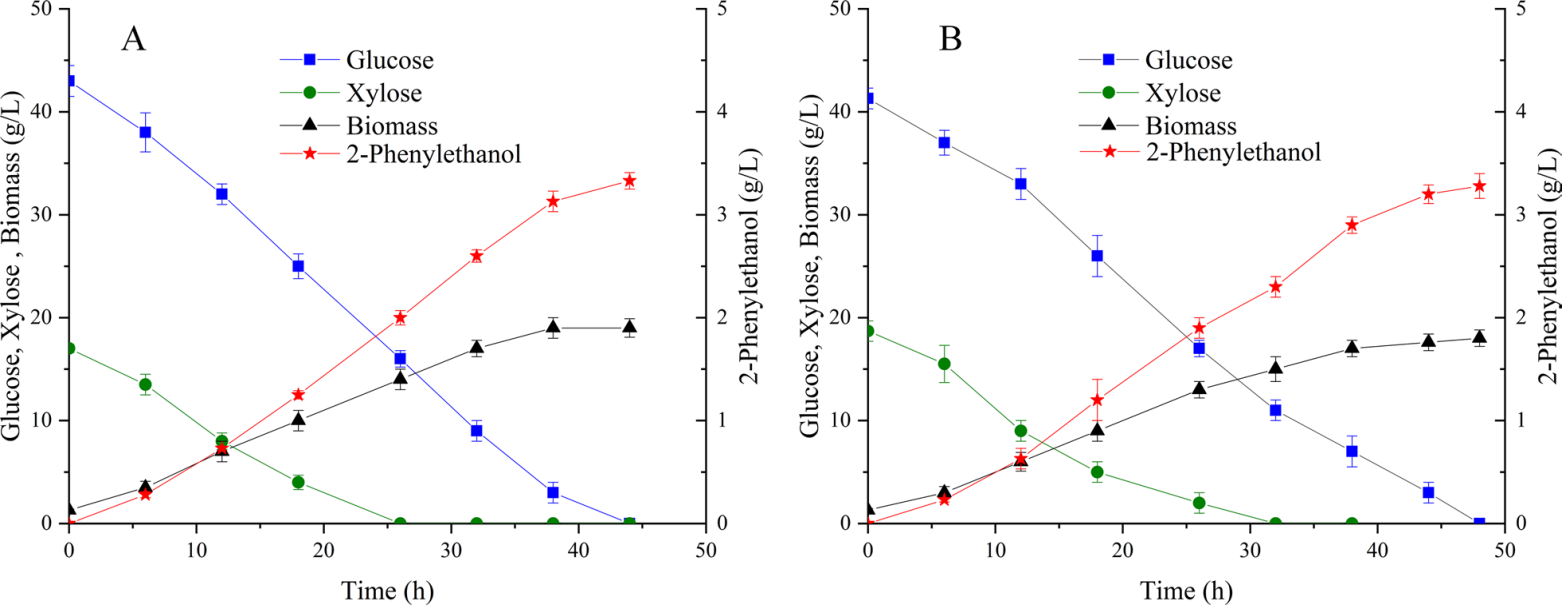
De novo production of 2-phenylethanol by *C. glutamicum* CGPE15 strain with mixture of glucose and xylose **A** and corn stalk hydrolysate **B**

Lignocellulose-treated sugars are usually mixed with microbial growth inhibitors, e.g., furans and phenolics [60, 61]. Suitable microbial hosts for bio-based chemicals production with these sugars should not only tolerate the toxicity of the final product, but also growth inhibitors from culture medium to achieve high titer and yield [19]. Therefore, the production of 2-phenylethanol and sugars utilization with a real hydrolysate were examined to evaluate its potential for practical application. The corn stalk hydrolysate treated with commercial Cellic CTec2 cellulase was used as feedstock. A total of 60 g/L sugar mixture containing 41.3 g/L glucose and 18.7 g/L xylose from corn stalk hydrolysate was adjusted to correspond to the cultivation with the single substrate. The analytical reagent sugar mixture was also used as the substrate for comparison. As shown in Fig. 5A, the engineered strain CGPE15 could effectively utilize the hydrolysate for 2-phenylethanol production, consuming glucose and xylose within 60 h. In addition, both sugars were simultaneously utilized at the start of the cultivation without a typical carbon catabolite repression. A similar result was observed for a glutamic acid-producing *C. glutamicum* expressing *xylAB* and *araE* when co-utilizing glucose and xylose [62]. The 2-phenylethanol titer of 3.28 g/L with corn stalk hydrolysate was obtained, which was comparable to that with analytical pure glucose and xylose of 3.33 g/L (Fig. 5B). There was no obvious difference in the carbon uptake manner between the cultivation with sugar mixture and corn stalk hydrolysate, although the growth with hydrolysate was slightly inhibited due to the presence of low contents of inhibitors. Even so, the selection of the robust strain *C. glutamicum* for 2-phenylethanol production showed an obvious advantage when lignocellulosic wastes were used as the feedstock considering its ability to withstand growth inhibitors and 2-phenylethanol.

## Conclusions

In this study, we established and validated an efficient *C. glutamicum* strain for the de novo production of 2-phenylethanol. The tolerance test of *C. glutamicum* indicated that the strain has high potential as a 2-phenylethanol production host. Mutations associated with the regulation of sigma factors were identified in both more tolerant evolved strains CALE1 and CALE2. Several KDCs and ADHs enzymes were screened to construct an Ehrlich pathway for the biosynthesis of 2-phenylethanol, and Aro10 originating from *S. cerevisiae* and YahK originating from *E. coli* were found to be the most efficient. 2-Phenylethanol production further increased after expressing key genes involved in the phenylpyruvate biosynthesis pathway. After engineering a xylose assimilation pathway, the final strain CGPE15 could produce 3.28 g/L 2-phenylethanol from a real and practical corn stalk hydrolysate. It is expected that 2-phenylethanol production could be further improved by reducing the by-products accumulation and optimizing the fermentation process. This study provides a promising route for commodity 2-phenylethanol bioproduction from lignocellulosic biomass.

## Supplementary Information

**Additional file 1: Table S1.** Primers used in this study. **Table S2.** Specific activity of the AdhA for phenylacetaldehyde and isobutyraldehyde. **Figure S1.** L-Phenylalanine production by *C. glutamicum*. **Figure S2.** Comparison of 2-phenylethanol production by *C. glutamicum* CGPE7 and CALE1(pEC-*aro10*).
