# Supplementary material for: Engineering Corynebacterium glutamicum for de novo production of 2-phenylethanol from lignocellulosic biomass hydrolysate
Source: Biotechnol Biofuels Bioprod. 2023 May 4;16:75. doi: 10.1186/s13068-023-02327-x (PMC10158149; doi:10.1186/s13068-023-02327-x)
Supplement: Supplementary file 1 — Additional file 1: Table S1. Primers used in this study. Table S2. Specific activity of the AdhA for phenylacetaldehyde and isobutyraldehyde. Figure S1. L-Phenylalanine production by C. glutamicum. Figure S2. Comparison of 2-phenylethanol production by C. glutamicum CGPE7 and CALE1(pEC-aro10). [file 13068_2023_2327_MOESM1_ESM.docx]

**Engineering** ***Corynebacterium glutamicum* for *de novo* production of** **2-****phenylethanol from** **lignocellulosic biomass hydrolysate**

Nianqing Zhu ^1^, Wenjing Xia ^1, 2,^ *, Guanglu Wang ^3^, Yuhe Song ^1^, Xinxing Gao ^1^, Jilei Liang ^1^, Yan Wang ^1^

*^1^ Jiangsu Key Laboratory of Chiral Pharmaceuticals Biosynthesis, College of Pharmacy and Chemistry & Chemical Engineering, Taizhou University, Taizhou 225300, Jiangsu, P.R. China*

*^2^ School of Chemistry and Biological Engineering, Nanjing Normal University Taizhou College,*

*Taizhou 225300, Jiangsu, P.R. China*

*^3^ Laboratory of Biotransformation and Biocatalysis, School of Food and Biological Engineering, Zhengzhou University of Light Industry, Zhengzhou, Henan 450000, P.R. China*

***^*^Corresponding author:*** ***Wenjing Xia,*** *E-mail address: xiawenjing@nnutc.edu.cn*

**Table S1.** Primers used in this study

| Oligonucleotide primers | Sequence | Purpose |
| --- | --- | --- |
| aro101 | AGTC**GAATTC***AAAGGAGGACAACC*ATGGCACCTGTGACC | overexpression of *aro10* |
| aro102 | CGGA**GGTACC**TTACTTCTTGTTCCTCTTCAGTGCTGCT | overexpression of *aro10* |
| pmkdc1 | GGCC**GAATTC***AAAGGAGGACAACC*ATGACCAACACTGTGA | overexpression of *pmkdc* |
| pmkdc2 | CGGC**GGTACC**TTAAAAAGAATACAAGGAGCTCTTGGAGC | overexpression of *pmkdc* |
| ipdc1 | GGCC**GAATTC***AAAGGAGGACAACC*ATGAAGTTGGCCGAA | overexpression of *ipdc* |
| ipdc2 | CGGC**GGTACC**TTATTCGCGTGGTGCCGCATGCAA | overexpression of *ipdc* |
| yahK1 | GCAC**GGTACC***AAAGGAGGACAACC*ATGAAGATCAAGGCAG | overexpression of *yahK* |
| yahK2 | CGGC**GGATCC** TTAATCAGTCAGGGTCCTATTATC | overexpression of *yahK* |
| yjgB1 | GAAC**GGTACC***AAAGGAGGACAACC*ATGCTGTACACCTCA | overexpression of *yjgB* |
| yjgB2 | ACGC**GGATCC**TTAGAAATCGGCTTTCAATACGACGCGG | overexpression of *yjgB* |
| adhA1 | TAAT**GGTACC***AAAGGAGGACAACC*ATGACCACTGCTGCACCCCAA | overexpression of *adhA* |
| adhA2 | ACGC**GGATCC**TTAGAAACGAATCGCCACACGACCATCGAT | overexpression of *adhA* |
| aroG^fbr^1 | TGCC**GGATCC**TTGACAATTAATCATCGGCTCG | overexpression of *aroG* |
| aroG^fbr^ 2 | ATCG**GTCGAC**TTATCCTCGTCGCGCCTTTACT | overexpression of *aroG* |
| aroH1 | TGCC**GGATCC**TTGACAATTAATCATCGGCTCG | overexpression of *aroH* |
| aroH2 | ATAA**GTCGAC**TTAGAAACGGGTGTCCACTGCG | overexpression of *aroH* |
| pheA^fbr^1 | GCAA**GTCGAC***AAAGGAGGACAACC*ATGACTTCCGAGAACCCTCT | overexpression of *pheA* |
| pheA^fbr^2 | CCCC**AAGCTT**TTATGTTGGGTCCACGGGGACAACATTCTCAGA | overexpression of *pheA* |
| aroL1 | CTAG**AAGCTT***AAAGGAGGACAACC*ATGTCGACTCACACATCTTCAAC | Overexpression of *aroL* |
| aroL2 | CTAGT**AAGCTT**TTAGGAAACGACGACGATCAAGT | Overexpression of *aroL* |
| aroA1 | CGGC**AAGCTT**C*AAAGGAGGACAACC*ATGGTCTTTGTGTCTGATTC | Overexpression of *aroA* |
| aroA2 | CGGC**AAGCTT**TTAGCCAACCATCTCCTCCCAAACA | Overexpression of *aroA* |
| ppsA1 | AGTC**AAGCTT**C*AAAGGAGGACAACC*ATGTCCAACAACGGCTCTAG | Overexpression of *ppsA* |
| ppsA2 | CGAA**GTCGAC**TTATTTTTTCAGCTCGGCTAGGCTCAGCC | Overexpression of *ppsA* |
| tkt1 | AGAA**GTCGAC**C*AAAGGAGGACAACC*ATGACCACCTTGACGCT | Overexpression of *tkt* |
| tkt2 | GCTA**GGATCC** TTAACCGTTAATGGAGTCCTTGGCC | Overexpression of *tkt* |
| P_hom_xylE1 | GAATCT**GGCGCC**CCGTTGAAAACTAAAAAGCTGG | Overexpression of *xylE* |
| P_hom_xylE2 | GAATCT**GGCGCC**TTATAAGGTTGCGGTTTGCTGAG | Overexpression of *xylE* |
| xylAB1 | AGCT**GGATCC**CCGACATCATAACGGTTCTG | Overexpression of *xylAB* |
| xylAB2 | AGCT**GAATTC**CCTTGGTACTGGGGGAGTGAT | Overexpression of *xylAB* |

The restriction sites are shown in bold, and RBS nucleotide sequences are shown in italics.

**Table S2.** Specific activity of the AdhA for phenylacetaldehyde and isobutyraldehyde.

| Strain | Substrate | Cofactor | Specific activity (U/mg) |
| --- | --- | --- | --- |
| CGPE4 | Phenylacetaldehyde | NADPH | 0.02±0.01 |
| CGPE4 | Isobutyraldehyde | NADH | 0.46±0.04 |
| CALE1(pEC-*aro10*) | Phenylacetaldehyde | NADPH | 0.03±0.01 |
| CALE1(pEC-*aro10*) | Isobutyraldehyde | NADH | 0.05±0.02 |

The values were given as the averages and standard deviations of three independent experiments.





**Figure S1. L-Phenylalanine production by *C. glutamicum*.** *C. glutamicum* strains were cultivated in 50 mL fermentation medium at 30 °C at an agitation of 200 rpm for 60 h. The fermentation medium was glucose (60 g/L), corn steep liquor (25 g/L), (NH_4_)_2_SO_4_ (15 g/L), urea (2 g/L), KH_2_PO_4_ (2 g/L), and MgSO _4_·7H_2_O (1 g/L), and pH7.0.





**Figure S2.** **Comparison of 2-phenylethanol production by *C. glutamicum* CALE1 CGPE7 and CALE1(pEC-*aro10*).** 2-Phenylethanol production was determined after 60 h fermentation in modified AY medium supplemented with 60 g/L glucose. The values were given as the averages and standard deviations of three independent cultures.
